# Gastric emptying in hereditary transthyretin amyloidosis: the impact of autonomic neuropathy

**DOI:** 10.1111/j.1365-2982.2012.01991.x

**Published:** 2012-12

**Authors:** J Wixner, P Karling, A Rydh, R Hörnsten, U Wiklund, I Anan, O B Suhr

**Affiliations:** * Departments of Public Health and Clinical Medicine, Umeå UniversityUmeå, Sweden; † Radiation Sciences and Diagnostic Radiology, Umeå UniversityUmeå, Sweden; ‡ Clinical Physiology, Heart Centre and Department of Surgical and Perioperative Science, Umeå UniversityUmeå, Sweden; § Radiation Sciences, Biomedical Engineering, Umeå UniversityUmeå, Sweden

**Keywords:** amyloidosis, autonomic nervous system disorders, gastric emptying, hereditary, nutrition status, transthyretin

## Abstract

**Background:**

Gastrointestinal (GI) complications are common in hereditary transthyretin amyloidosis and an autonomic dysfunction has been considered to explain these symptoms. The aim of this study was to investigate the impact of autonomic neuropathy on gastric emptying in hereditary transthyretin amyloidosis and to relate these findings to nutritional status, GI symptoms, gender, and age at disease onset.

**Methods:**

Gastric emptying was evaluated with gastric emptying scintigraphy. Spectral analysis of the heart rate variability and cardiovascular responses after tilt test were used to assess the autonomic function. The nutritional status was evaluated with the modified body mass index (s-albumine × BMI).

**Key Results:**

Gastric retention was found in about one-third of the patients. A weak correlation was found between the scintigraphic gastric emptying rate and both the sympathetic (*rs* = −0.397, *P* < 0.001) and parasympathetic function (*rs* = −0.282, *P* = 0.002). The gastric emptying rate was slower in those with lower or both upper and lower GI symptoms compared with those without symptoms (median T_50_ 123 *vs* 113 min, *P* = 0.042 and 192 *vs* 113 min, *P* = 0.003, respectively). Multiple logistic regression analysis showed that age of onset (OR 0.10, CI 0.02–0.52) and sympathetic dysfunction (OR 0.23, CI 0.10–0.51), but not gender (OR 0.76, CI 0.31–1.84) and parasympathetic dysfunction (OR 1.81, CI 0.72–4.56), contributed to gastric retention.

**Conclusions and Inferences:**

Gastric retention is common in hereditary transthyretin amyloidosis early after onset. Autonomic neuropathy only weakly correlates with gastric retention and therefore additional factors must be involved.

Hereditary transthyretin amyloidosis or familial amyloidotic polyneuropathy (FAP) is a dominantly inherited transthyretin amyloidosis that is caused by mutated transthyretin (TTR). There are approximately 100 known amyloidogenic transthyretin (ATTR) mutations of which ATTR Val30Met (methionine substituted for valine at position 30) is the most common, leading to a neuropathic form of the disease, FAP.^1^ FAP Val30Met is present all over the world with endemic areas in Portugal, Brazil, Sweden, and Japan. Symptoms are caused by deposition of amyloid fibrils in various body tissues and include, for example, peripheral polyneuropathy, autonomic neuropathy, cardiac arrhythmias, and gastrointestinal (GI) disturbances. Virtually all patients develop GI complications during the course of the disease^2^ and the initial constipation is later relieved by bursts of diarrhea that successively become permanent. Nausea and vomiting are also reported by many patients.

Without treatment the average survival in Sweden is 9–13 years after onset^2^,^3^ and death is caused by severe malnutrition and opportunistic infections in many cases.^3^,^4^ As nearly all circulating TTR is produced by the liver, a liver transplantation (Ltx) that ceases the synthesis of mutated TTR has proved to halt the progression of the disease. The GI disturbances of FAP patients lead to malnutrition, which negatively affects the outcome of Ltx,^5^ and are hence important for morbidity and mortality after the procedure.

The mechanisms behind the GI complications in FAP are poorly understood, but it has been suggested that an autonomic neuropathy is at least partly responsible.^4^ The aim of the present investigation was to assess the occurrence of gastric retention in FAP patients and relate the findings to autonomic function measured by heart rate variability (HRV) and tilt test. We also wanted to relate the findings to the patients’ nutritional status measured by the modified body mass index (mBMI), GI symptoms, gender, and age at disease onset.

## Materials and Methods

### Patients

One hundred and eighty-eight Swedish FAP patients were available for the study (Table 1). All patients were examined at the Department of Medicine, Umeå University Hospital, Umeå, Sweden, between 1990 and 2009 as part of an investigation of their disease and, for the majority of the patients, also as part of the evaluation for Ltx. The FAP diagnosis was based on clinical findings consistent with FAP, presence of amyloid fibrils in an intestinal, skin, or abdominal fat biopsy, and identification of an amyloidogenic TTR (ATTR) mutation. Practically all patients carried the Val30Met mutation except 6 who carried the Leu55Gln, Phe33Leu, Tyr69His, His88Arg, Gly57Arg, and Val30Leu mutations, respectively. Clinical data were obtained from the first evaluation after onset of the disease. One hundred and forty-one patients (75%) were still alive at the time of the study (December 2009). For each patient all examinations were carried out within a 6 months period of time.

**Table 1:** Clinical data of the patients

	All patients	Patients with T_50_ ≥ 350 min
Total number of patients (men/women)	188 (117/71)	13 (8/5)
Median age at onset (range)	57 (22–82) years	63 (31–70) years
Median duration of disease (range)	3 (0–16) years	4 (1–12) years
Number of patients with FAP Val30Met	182 (97%)	12 (92%)
Number of patients with GI symptoms	111 (59%)	10 (77%)
Upper (nausea/vomiting)	14 (13%)	0 (0%)
Lower (constipation/diarrhea)	83 (74%)	8 (62%)
Both upper and lower	14 (13%)	2 (15%)

FAP, familial amyloidotic polyneuropathy; Val30Met, transthyretin mutation where methionine is substituted for valine at position 30; GI, gastrointestinal; T_50_, total half-life for the radioactive marker in the gastric emptying scintigraphy, values above 350 min are consistent with a severely delayed gastric emptying.

### Gastric emptying scintigraphy (GES)

In 162 (86%) of the patients a GES was performed. Twenty-six patients did not undergo GES, nine because it was not available at the time of the examination, and 17 because of technical problems at the scheduled time of examination. The measurement was carried out according to the method employed in the Swedish multicenter study of gastric emptying.^6^ The scintigraphic acquisitions were performed using a STARCAM gamma camera (General Electric, Milwaukee, WI, USA) with a low energy, general-purpose collimator and a 128 × 128 matrix.

**Figure 1:**
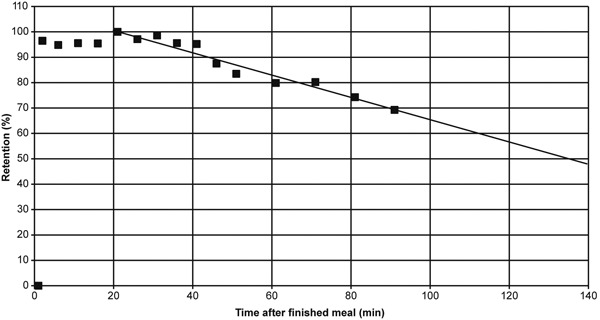
Gastric emptying scintigraphy. Graph showing the result from a slightly delayed gastric emptying scintigraphy (136 min) in a 54-year-old female patient with hereditary transthyretin amyloidosis. Lag phase from 0 to 20 min.

The collected data were then electronically plotted on a graph (Fig. 1) that was printed and manually analyzed by one examiner (JW) who calculated the lag phase, T_50_, and T_1/2_ (T_50_ being the total half-life and T_1/2_ the half-life after the lag phase). Abnormal gastric emptying was defined as a T_50_ above 133 min (mean + 2 SD) according to the reference values obtained by the Swedish national multicenter study of 160 healthy individuals aged 17–80 years.^6^ T_50_ over 350 min was entered as 350 min.

### Nutritional status

The patients’ nutritional status was assessed by the mBMI, in which BMI (kg m^−2^) is multiplied by serum albumin (g L^−1^) to compensate for edema.^3^ mBMI could be calculated in 185 (98%) of the patients. Values below 750 were considered consistent with underweight and values below 600 were regarded as consistent with severe malnutrition.^3^,^5^

### HRV

**Figure 2:**
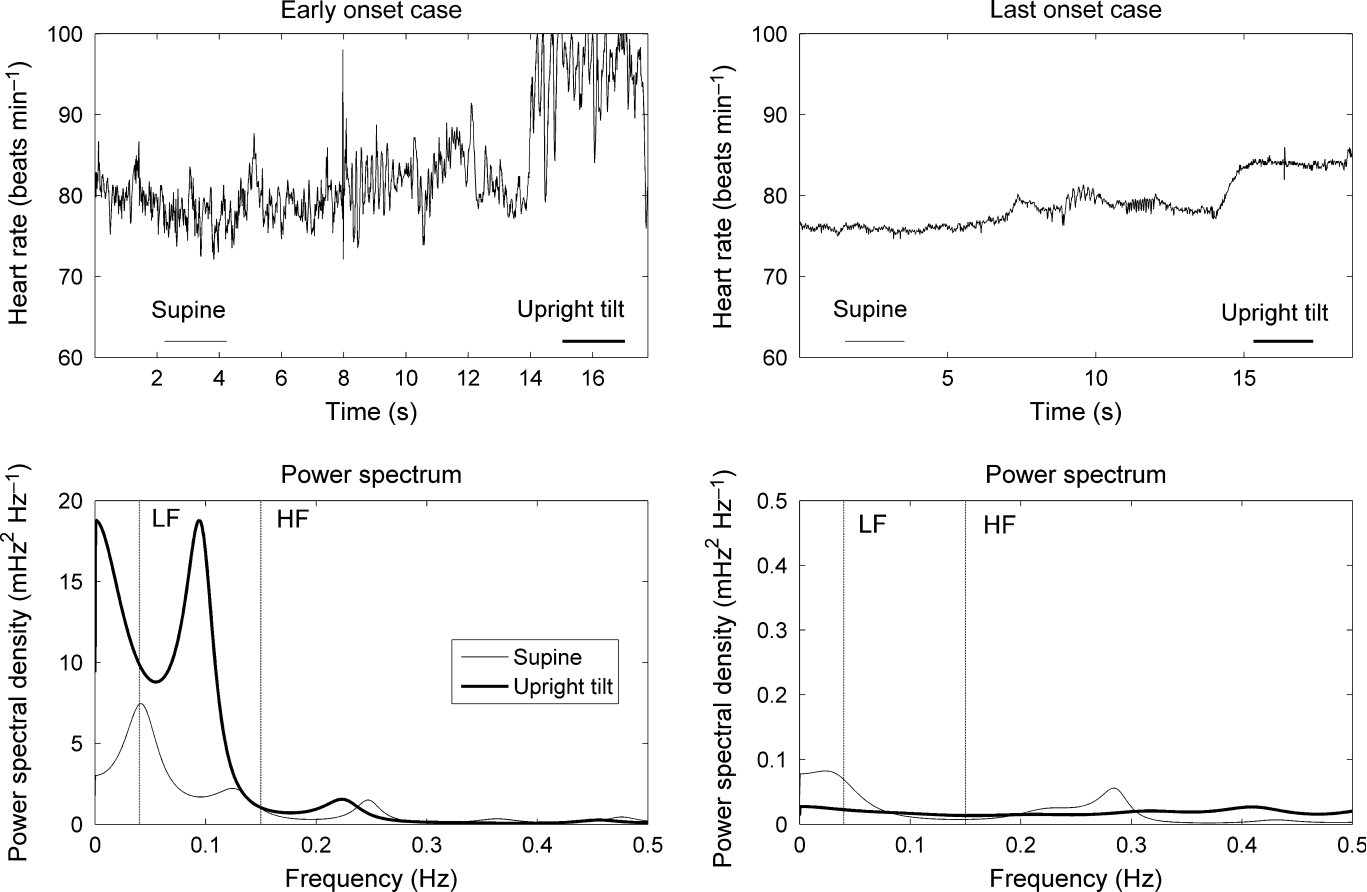
Heart rate variability (HRV). Heart rate and spectral analysis of the HRV in two patients with hereditary transthyretin amyloidosis. To the left an early onset case with an almost normal HRV and to the right a late onset case with a low HRV. The heart rate was measured in both the supine and the upright position. The high-frequency component in the supine position (HF_sup_) was used as an indirect estimate of the parasympathetic function and the low-frequency component in an upright position (LF_tilt_) as an estimate of the sympathetic function.

Heart rate variability was recorded in 177 (94%) of the patients. The power spectral analysis was estimated by auto-regressive modeling, consequently using Burg-algorithm with 30 parameters.^7^ The spectral power in two frequency bands was used in the investigation – the high-frequency component (0.15–0.50 Hz) recorded in a supine position (HF_sup_) and the low-frequency component (0.04–0.15 Hz) recorded in an upright position (LF_tilt_) as presented in Fig. 2. The respiration-related high-frequency component of HRV represents an indirect estimate of vagal cardiac control and the low-frequency component, recorded after postural change from a supine to an upright position, is a useful marker of sympathetic activity.^8^,^9^ All frequency-domain HRV indices were log transformed (base 10) because of skewed distribution. As the heart rate was expressed in mHz and as spectral power corresponds to the variance, all spectral indices had the unit mHz^2^, but became dimensionless after log transformation. All patients with pacemaker treatment and frequent non-sinus beats were excluded from the analysis, where patients with non-neurogenic HRV patterns were identified by comparing with power spectra for respiration and by inspection of the pattern in the beat-to-beat fluctuations in R-R intervals.^10^–^14^

### Tilt test

During the HRV examinations the blood pressure in the right upper arm was measured with cuff and stethoscope after 3 min in the supine resting position and after 3 min in the tilted (70°) upright position. The mean heart rate (HR) was calculated from the 2 min ECG recordings used for the HRV analysis, of which all were adjacent to the measurements of the blood pressure. The changes in systolic blood pressure (SBP) and mean HR after tilting were then calculated. Data on changes in SBP and mean HR were recorded in 167 (89%) and 138 (73%) of the patients, respectively. A decrease in SBP of 20 mm Hg or more was regarded as consistent with orthostatic hypotension.^15^

### Statistical analysis

Statistical analyses were, if possible, performed by non-parametrical methods. Differences between groups were tested with the Mann–Whitney *U* test, Kruskal–Wallis test, and the chi-squared test. Correlation was analyzed with Spearman’s rank order test. The relationship between variables was tested with logistic regression. *P* values below 0.05 were regarded as statistically significant. PASW Statistics 18 for Macintosh was used for the calculations.

### Ethics

The study is part of a larger project that is approved by the Regional ethics board in Umeå, Sweden; reference number 06–084M.

## Results

### GI symptoms and nutritional status

Data on GI symptoms were available for all patients except one. Fifty-nine percent suffered from GI disturbances, and median mBMI was 961 (range 550–1535). In 181 (98%) of the patients the mBMI was 600 or more, and in 4 (2%) it was below 600.

All patients with severe malnutrition were women and all of them had GI symptoms. Patients with mBMI below 600 had a lower age at onset (47 *vs* 57 years), a longer duration of disease (10 *vs* 3 years), and lower LF_tilt_ (1.33 *vs* 2.08) and HF_sup_ (1.55 *vs* 1.77) than those with mBMI of 600 or more. However, the number of patients with severe malnutrition was too small for adequate statistical calculations.

### Gastric emptying

Gastric emptying scintigraphy disclosed gastric retention in 63 of 162 patients (39%). Median T_50_ was 119 (range 48–350) min. In 13 patients the gastric emptying was severely delayed with T_50_ of 350 min or more.

A small difference in T_50_ was found between men and women, with the latter showing a slower gastric emptying rate (median T_50_ 116 *vs* 128 min, *P* = 0.043). No significant correlation was found between the age of onset and T_50_ (*rs* = −0.026, p = 0.744). There was a weak but significant negative correlation between T_50_ and mBMI (*rs* = −0.218, *P* = 0.006).

### Gastric emptying and GI symptoms

**Figure 3:**
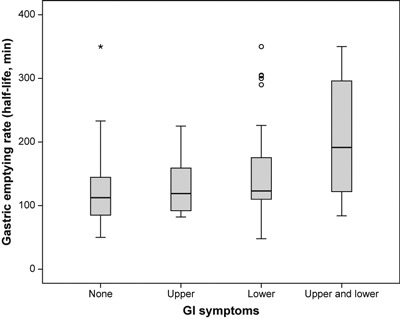
Gastrointestinal symptoms and gastric emptying. Box plot showing the differences in the scintigraphic gastric emptying rate related to gastrointestinal (GI) symptoms (*P* = 0.004). Upper GI symptoms = nausea/vomiting. Lower GI symptoms = diarrhea/constipation.

When comparing the reported GI symptoms with the outcome of GES, we found significant differences in T_50_ between the groups (Fig. 3). Post hoc analysis showed the strongest significance between patients without symptoms compared to those with both upper and lower GI symptoms (median T_50_ 113 *vs* 192 min, *P* = 0.003). A significant difference in T_50_ between patients without and those with lower GI symptoms (median T_50_ 113 *vs* 123 min, *P* = 0.042) was also found. No significant difference was found between patients without and those with upper GI symptoms (median T_50_ 113 *vs* 119 min, *P* = 1).

### Autonomic function

The spectral analysis of HRV could be performed in 134 (76%) of the patients. In 23 (13%) of the patients arrhythmia precluded the analysis of HRV and in 20 (11%) patients data were missing or not applicable (due to pacemakers or data file errors). The median HF_sup_ was 1.77 (range 0.11–3.49) and the median LF_tilt_ was 2.07 (range 0.02–4.17). In comparison, healthy subjects registered in our database of healthy volunteers had a median HF_sup_ of 2.52 (range 1.16–4.24) and a median LF_tilt_ of 2.98 (range 0.00–4.06).

Significant differences in HRV between female and male patients were found for HF_sup_ (median HF_sup_ 1.98 *vs* 1.67, *P* = 0.008) and LF_tilt_ (median HF_sup_ 2.28 *vs* 1.89, *P* = 0.015), respectively. Negative correlations between the age of onset and HF_sup_ (*rs* = −0.194, *P* = 0.025) and LF_tilt_ (*rs* = −0.395, *P* < 0.001) were found, where older patients displayed a lower HRV.

No significant correlation was found between HF_sup_ and mBMI (*rs* = 0.147, *P* = 0.089), but there was a correlation between LF_tilt_ and mBMI (*rs* = 0.280, *P* = 0.001). No significant difference in HF_sup_ (*P* = 0.822) or LF_tilt_ (*P* = 0.765) was seen between patients without GI symptoms or those with upper (median HF_sup_ 1.76 *vs* 1.78, median LF_tilt_ 2.10 *vs* 1.83), lower (median HF_sup_ 1.76 *vs* 1.74, median LF_tilt_ 2.10 *vs* 2.08), or both upper and lower GI symptoms (median HF_sup_ 1.76 *vs* 1.58, median LF_tilt_ 2.10 *vs* 1.71).

### Autonomic function and gastric emptying

**Figure 4:**
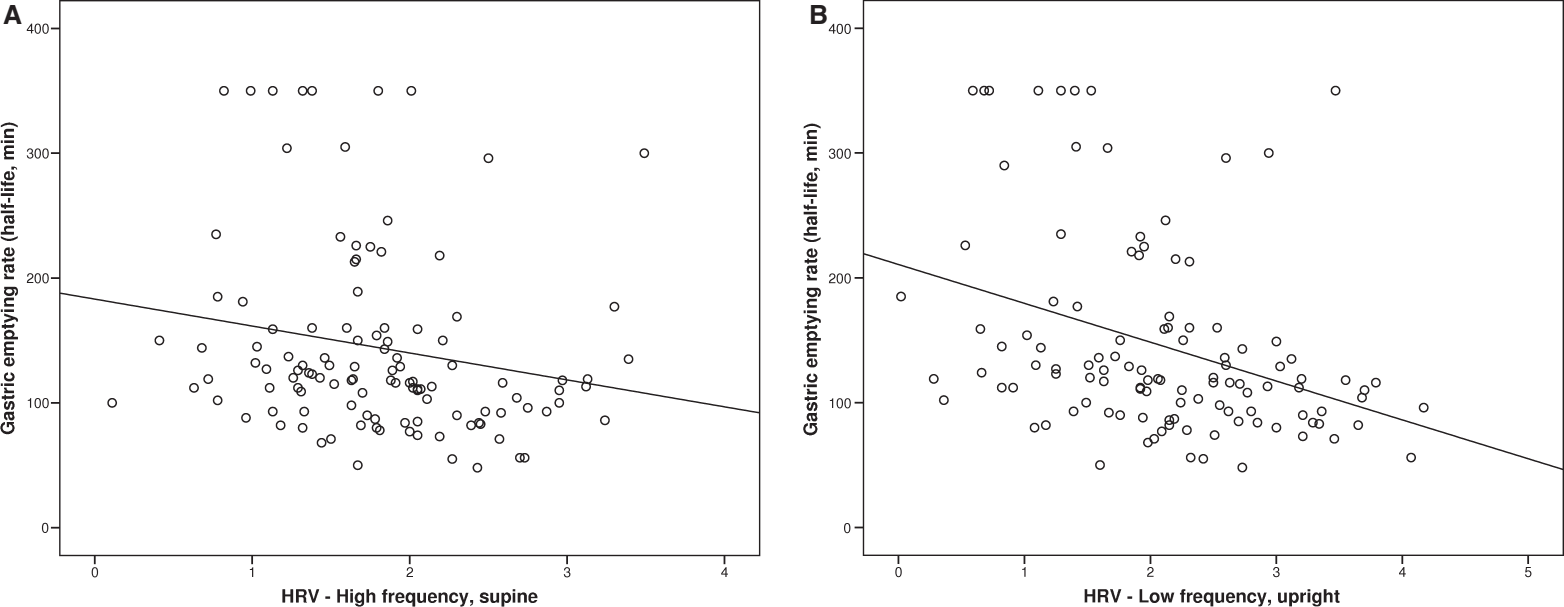
Autonomic function and gastric emptying. Scatterplot showing the relationship between (A) the scintigraphic gastric emptying rate and the parasympathetic function (*rs* = −0.282, *P* = 0.002) and (B) the relationship between the scintigraphic gastric emptying rate and the sympathetic function (*rs* = −0.397, *P* < 0.001). HRV = heart rate variability.

Weak but significant correlations were found between T_50_ and HF_sup_ (Fig. 4A) and T_50_ and LF_tilt_ (Fig. 4B). Patients with retention at GES had a greater decrease in SBP after tilting than those without retention (median ΔSBP −10 *vs*−5 mm Hg, *P* = 0.038). No difference in the change in HR after tilting was found between patients with or without retention at GES (median ΔHR 7.56 *vs* 9.06 beats/min, *P* = 0.535). Retention at GES was significantly more common in patients with orthostatic hypotension than in those with no orthostatic hypotension (χ^2^ = 6.66, *P* = 0.010).

### Characteristics of patients with severe gastric retention

To further assess the possible mechanisms behind the GI complications, subgroup analyses were performed for patients with T_50_ of 350 min or more. Patients’ details are outlined in Table 1.

Patients with a severely delayed gastric emptying had significantly lower LF_tilt_ than those with T_50_ below 350 min (median LF_tilt_ 1.20 *vs* 2.14, *P* = 0.010) and they also had lower HF_sup_, however, this difference was not statistically significant (median HF_sup_ 1.35 *vs* 1.79, *P* = 0.063). Orthostatic hypotension was significantly more common in patients with severe gastric retention (χ^2^ = 5.74, *P* = 0.017).

A significant difference was also found for mBMI, where those with severely delayed gastric emptying showed lower mBMI than those without (median mBMI 809 *vs* 961, *P* = 0.004). No significant difference was found in age of disease onset (median age at onset 63 *vs* 56 years, *P* = 0.075) between patients with or without severe gastric retention. The number of patients was too small for valid statistical analyses on differences related to gender and GI symptoms.

### Multiple logistic regression analysis on factors behind gastric retention

To identify factors with an impact on gastric retention, HF_sup_, LF_tilt_, gender, and age at disease onset were utilized as independent factors in multiple logistic regression analyses. The age at onset and LF_tilt_ were the only factors significantly contributing to gastric retention. Details are outlined in Table 2.

**Table 2:** Multiple logistic regression analysis

	Univariate (crude OR)	Multivariate (adjusted OR)
Gender (female reference)	1.40 (CI 0.74–2.67)	0.76 (CI 0.31–1.84)
Age at disease onset (log)	0.74 (CI 0.25–2.22)	0.10 (CI 0.02–0.52)*
Parasympathetic function	0.58 (CI 0.32–1.06)	1.81 (CI 0.72–4.56)
Sympathetic function	0.45 (CI 0.27–0.74)*	0.23 (CI 0.10–0.51)*

OR, odds ratio; CI, 95% confidence interval; Log, log transformed because of skewed distribution.

Gastric retention determined with gastric emptying scintigraphy.

* Statistically significant.

## Discussion

To the best of our knowledge, this is the first and largest study on FAP patients exploring the impact of autonomic dysfunction on GI symptoms and gastric emptying. The study showed a high prevalence of delayed gastric emptying and also that delayed gastric emptying is a common feature even early after disease onset. In consistency, unpublished study data from upper GI endoscopies after an overnight fast showed that 29% of the patients had retention of undigested food.

Contrary to our expectations, only a weak correlation between delayed gastric emptying and autonomic neuropathy was found. Surprisingly, sympathetic activity appeared to be strongly, or at least similarly, related to gastric emptying as parasympathetic activity. This was also supported by a higher frequency of gastric retention in patients with orthostatic hypotension and by subgroup analyses for patients with severely delayed gastric emptying.

Parasympathetic dysfunction often precedes sympathetic dysfunction in FAP patients.^16^ The fact that the sympathetic function was more strongly correlated with gastric retention in our study probably reflects a more advanced disease in patients with sympathetic dysfunction. In the multiple logistic regression analysis, sympathetic dysfunction significantly contributed to gastric retention, whereas the parasympathetic did not, indicating that an intact parasympathetic function is not the only factor important for a preserved gastric emptying rate. A case report from Ohio has also shown that gastroparesis can occur in patients with pure sympathetic dysfunction,^17^ suggesting that a reduced sympathetic function itself may negatively affect gastric emptying.

As vagotomy (i.e., loss of parasympathetic control) often leads to gastric retention,^18^ and as there is evidence of amyloid deposits in the autonomic nervous system with a destruction of the vagal nerve in FAP patients,^19^ an autonomic neuropathy has been suggested to be the underlying factor for GI disturbances of these patients.^20^ This is also supported by the findings of a destructed celiac ganglion in FAP patients,^21^ but our findings of delayed gastric emptying in patients with normal sympathetic and parasympathetic activity implies that additional factors must be involved.

Such possible contributing factors may be a depletion of the GI neuroendocrine cells and a destruction of the enteric nervous system.^22^–^25^ However, the enteric nervous system seems to be unaffected in FAP Val30Met^26^,^27^ and no improvement of the GI function has been shown after Ltx,^28^,^29^ although a normalization of the endocrine cell count was noted.^30^ In diabetes mellitus a down-regulation of ghrelin and its receptor is linked to GI dysfunction,^31^,^32^ and it would be of interest to study if this is also the case in FAP patients.

Likewise, a depletion of the interstitial cells of Cajal (ICC), increased oxidative stress, and smooth muscle degeneration and fibrosis play important roles in the gastroenteropathy of diabetes mellitus,^33^–^35^ and need to be investigated in FAP as well. Preliminary results from another of our studies in fact showed a marked decrease of gastric ICC in FAP patients compared with controls. Interestingly, an accumulation of advanced glycation end products, which bind to enteric neurons and cause a decreased production of nitric oxide synthase leading to a delayed gastric emptying in diabetics,^34^ have also been observed in FAP.^36^ Analysis of urinary secreted NO_2_^−^/NO_3_^−^ levels has implicated a decreased NO synthesis in FAP patients.^37^

Nausea, vomiting, and early satiety are classic symptoms of gastroparesis. Unexpectedly, none of the patients with a severely delayed gastric emptying reported upper GI symptoms. Furthermore, there was no difference in the outcome of GES when comparing patients without symptoms with those with upper GI symptoms, whereas lower GI symptoms and, especially, a combination of upper and lower GI symptoms seemed to be more related to gastric retention in FAP. A possible explanation may be that those patients have a more pronounced GI dysfunction and it appears to be difficult to predict the presence of gastroparesis from GI symptoms alone.^38^

Delayed gastric emptying correlated with a low mBMI and patients with severe gastric retention had significantly lower mBMI compared with those with T_50_ below 350 min. Previous studies have shown that GI dysfunction and a low mBMI are predictors of an increased mortality after Ltx, as is an early onset of GI disturbances.^3^,^5^ Thus, the detection of gastric retention is an important part of the evaluation of FAP patients, and GES is therefore part of our routine investigation of patients under evaluation for Ltx.

We noted that the female patients displayed higher HF_sup_ and LF_tilt_ and slower gastric emptying rates than males and that the group of patients with severe malnutrition consisted of women only. However, no gender related differences in survival after Ltx have been observed, except for a decreased survival of late onset males.^39^ We do not believe that the gender differences observed have any clinical impact.

Expectedly, a weak negative correlation was found between age at onset and HF_sup_ and LF_tilt_. A decline in the autonomic nervous function is also found in the general population and HRV values are often age adjusted. The values displayed in the present investigation were, however, not age adjusted. A late onset of the disease also significantly contributes to gastric retention, probably reflecting a poorer autonomic function.

In conclusion, hereditary TTR amyloidosis was associated with delayed gastric emptying early after disease onset in Swedish patients. Furthermore, gastric retention is associated with a poorer nutritional status. Only weak correlations were found between the measures of autonomic neuropathy and gastric retention, indicating that other factors must be involved. Surprisingly, no correlation was found between upper GI symptoms and delayed gastric emptying, suggesting that GI symptoms are poor predictors of the actual GI function in these patients.

### Limitations

The HF_sup_ and LF_tilt_ from spectral analysis of HRV were used as markers of parasympathetic and sympathetic autonomic function, respectively, as they are used as estimates of autonomic cardiac control and one may argue that they do not fully correspond to the autonomic gastric control. However, HRV is generally considered to be a good marker for autonomic function in FAP^20^ and has also been used in studies on autonomic function and GI motility in diabetics.^40^,^41^

A scintigraphic measurement of 4 h would improve the accuracy,^42^ but a 2-h measurement is the standard procedure at our hospital reflecting current clinical practice. The manual measurement of the lag phase, T_50_, and T_1/2_ by a single reviewer has an advantage over the automatic version because the settings of the latter method have been changed over the years making it less homogeneous. However, the manual analysis may not be as precise as the automatic.

## Funding

This study was supported by grants from the Swedish Heart and Lung Foundation, the patients organizations FAMY/AMYL in Västerbotten and Norrbotten, Umeå University, the 6th research framework of EU, The Euramy project, ALF-grants from Umeå University Hospital, and a ‘Spearhead’ grant from Västerbotten’s county.

## Disclosure

The authors have no competing interests.

## Author Contribution

Co-author OBS examined the patients, designed the research study, contributed to the interpretation of the results, and revised the manuscript; JW analyzed the data and wrote the manuscript; UW and RH provided and analyzed the HRV examinations and revised the manuscript; AR provided the GES examinations and revised the manuscript; PK helped with the interpretation of the results, the statistical analysis, and the revision of the manuscript; IA contributed to the interpretation of the results and revised the manuscript.

## Abbreviations

ATTR Val30Met: amyloidogenic transthyretin mutation where methionine is substituted for valine at position 30
ATTR: amyloidogenic transthyretin
BMI: body mass index
CI: confidence interval
FAP: familial amyloidotic polyneuropathy
GES: gastric emptying scintigraphy
GI: gastrointestinal
HF_sup_: high-frequency component of HRV in a supine position, i.e., parasympathetic function
HR: heart rate
HRV: heart rate variability
ICC: interstitial cells of Cajal
LF_tilt_: low-frequency component of HRV in an upright position, i.e., sympathetic function
Ltx: liver transplantation
mBMI: modified body mass index (s-albumine x BMI)
nNOS: nitric oxide synthase
NO: nitric oxide
OR: odds ratio
SBP: systolic blood pressure
T_½_: half-life for the radioactive marker after the lag phase in GES
T_50_: total half-life for the radioactive marker in GES
TTR: transthyretin
